# Recontorneo estético funcional míninamente invasivo mediante reducción selectiva del esmalte. Un reporte de caso

**DOI:** 10.21142/2523-2754-1303-2025-257

**Published:** 2025-08-31

**Authors:** Pablo Sanabria

**Affiliations:** 1 Postgrado en Prostodoncia, Universidad El Bosque. Bogota, Colombia drsanabria@dentaldag.com Universidad El Bosque Universidad El Bosque Bogota Colombia drsanabria@dentaldag.com

**Keywords:** esmalte dental, odontología estética, operatoria dental, reporte de caso, dental enamel, esthetics, dental, dentistry, operative, case report

## Abstract

La odontología estética es cada vez más demandada por los pacientes. En algunos casos requiere modificaciones mínimas del tamaño, forma o proporciones de los dientes, para lo cual existen principalmente técnicas aditivas directas o indirectas. De la misma manera, la odontología conservadora busca cumplir objetivos estéticos y funcionales con una mínima intervención. El caso clínico presentado describe un recontorneado estético funcional mínimamente invasivo con reducción selectiva del esmalte de los dientes anteriores, a fin de optimizar los resultados estéticos de un paciente masculino de 30 años que consultó por una mejoría estética en la apariencia de su sonrisa. Esta técnica ofrece ventajas como resultados inmediatos con beneficios estéticos y funcionales que pueden considerarse económicos y seguros comparados con los métodos convencionales.

## Abstract (en)

Aesthetic dentistry is increasingly in demand by patients, in some cases, minimal modifications to the size, shape, or proportions of teeth are required, for which there are mainly direct or indirect additive techniques. Similarly, conservative dentistry seeks to meet aesthetic and functional objectives with minimal intervention. This clinical case describes a minimally invasive functional aesthetic recontouring with selective reduction of the enamel of the anterior teeth to optimize the aesthetic results of a 30-year-old male patient who consulted for an aesthetic improvement in the appearance of his smile. This technique offers advantages such as immediate results with aesthetic and functional benefits that can be considered economical and safe compared to conventional methods.

## INTRODUCCIÓN

Los defectos físicos pueden llegar a ser muy influyentes en la autoestima o autopercepción de las personas. La satisfacción con la apariencia dental se puede ver afectada por el color, tamaño y forma de los dientes, la sensación de apiñamiento de estos o incluso el deseo de restauraciones estéticas posteriores al tratamiento de ortodoncia ^1^. Tradicionalmente, se ha considerado que los patrones estéticos dentales incluyen una sonrisa que muestra dientes blancos en armonía con los tejidos blandos. Sin embargo, los tratamientos odontológicos con fines estéticos deben planificarse de forma personalizada y así tener una guía para mejorar la estética ^2^.

Han surgido algunas técnicas alternativas para modificar los contornos de los dientes, entre ellas el recontorneo cosmético. Estas se pueden lograr mediante una técnica aditiva con resina compuesta, lo que permite un tratamiento conservador con un bajo costo biológico y financiero ^3^. Adicionalmente, la resina compuesta y las carillas de cerámica han demostrado tasas de supervivencia moderadas y altas para contornos modificados de dientes anteriores. No obstante, se han reportado algunas complicaciones biológicas, entre ellas problemas endodónticos, caries recurrentes, sensibilidad posoperatoria, desgaste y fractura dentaria. También se han reportado complicaciones mecánicas o estéticas, particularmente rugosidad de la superficie, cambios de color y decoloración marginal ^4^^,^^5^.

Por otra parte, los métodos sustractivos para recontornear la forma del diente incluyen el alargamiento de la corona y el modelado biológico. Este último es una técnica quirúrgica que preserva el hueso mientras establece la unión del tejido supracrestal necesaria para la restauración de los dientes. Eventualmente, ayuda a mejorar las medidas de higiene tanto para los pacientes como para los profesionales dentales, y crea un entorno biocompatible necesario para el éxito a largo plazo ^6^. Desafortunadamente, es usual la falta de información sobre las técnicas sustractivas en las superficies de los dientes para remodelar las piezas anteriores.

Como alternativa al recontorneo de las superficies interproximales de los dientes en pacientes con apiñamiento moderado, Sheridan propuso el *air-rotor stripping* (ARS), el cual podría ser una opción para las superficies vestibulares teniendo en cuenta que se estudiaron los efectos sobre el perfil facial con efectos positivos ^7^^,^^8^. La presente técnica describe un recontorneo basado en la reducción selectiva del esmalte de los dientes anteriores naturales con beneficios altamente estéticos; asimismo, esta técnica eventualmente podría usarse bucalmente.

## REPORTE DE CASO

Antes de realizar el procedimiento, el paciente firmó un consentimiento informado. Los requerimientos específicos para desarrollar esta técnica son limitados en cuanto al análisis de la selección de casos; por ello es tan importante en la valoración clínica tener muy claro el alcance de la técnica en cuanto al resultado estético final. Los pasos que se deben seguir son detallados a continuación.

Se seleccionó al paciente con base en características clínicas específicas, siendo su mayor deseo una excelente estética dental. Estos son los criterios de selección para el uso de esta técnica: movimientos dentales mínimos como rotaciones y extrusiones mínimas, requerimientos estéticos mínimos posortodoncia, movimientos funcionales como guía anterior y canina sin interferencias, maloclusión dental clase I y II, salud periodontal, dientes sin defectos de desarrollo del esmalte, buen estado de restauraciones preexistentes en el sector anterior.

**Figura 1:**
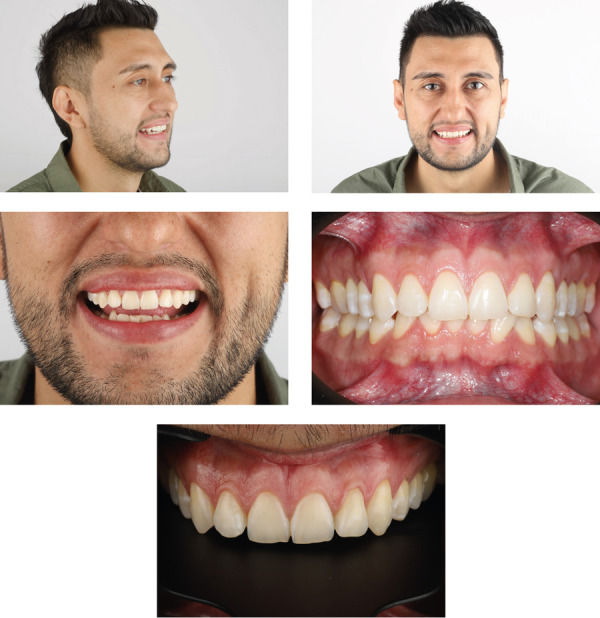
Registro fotográfico: a) Imagen lateral a 45° (sonrisa derecha), b) Imagen de sonrisa frontal, c) Imagen de sonrisa frontal en inoclusión, d) Imagen frontal en oclusión, e) Dientes anteriores superiores

Para tener la mayor cantidad de información que le ayude a planificar el proceso, se evaluó tanto fotografías intra como extraorales, las cuales incluyen las siguientes fotos: a) Imagen lateral de 45° (derecha, sonrisa), b) Imagen frontal de sonrisa, c) Imagen frontal de sonrisa facial sin oclusión, d) Imagen frontal de no oclusión, e) Imagen frontal de dientes anterosuperiores (Fig. 1).

**Figura 2:**
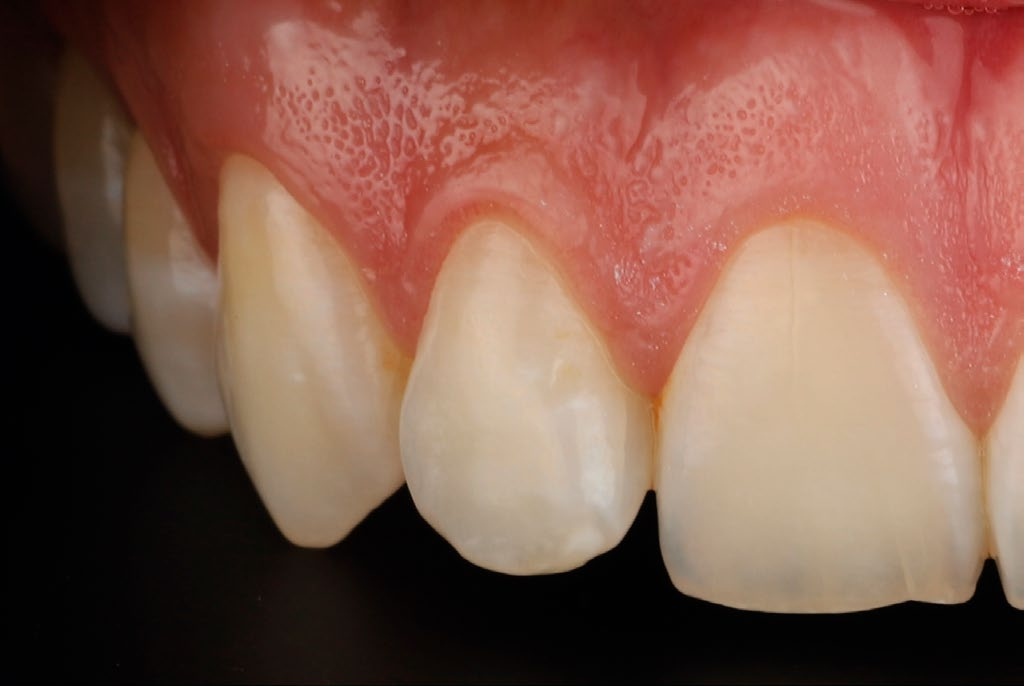
Análisis estético del esmalte.

Se evaluó la sonrisa desde diferentes ángulos, considerando algunos aspectos como el habla, el gesto, los labios en reposo y el movimiento para determinar la posible longitud de los dientes en los diferentes movimientos de sonrisa, también analizaron las líneas de sonrisa y la dominancia de los dientes anteriores. Se realizó un análisis del estado periodontal que incluye una evaluación de la salud gingival, papilas interdentales, márgenes gingivales, ancho de encía queratinizada, posiciones cenitales gingivales y su relación con la sonrisa y el habla, color de la mucosa y fenotipo periodontal. Adicionalmente, se realizó un análisis del tejido duro dental existente donde se evaluó continuidad del esmalte, brillo, translucidez, defectos del esmalte y textura (Fig. 2). 

Posteriormente se evaluó la oclusión dinámica. Debe existir equilibrio oclusal, muscular y articular, es decir, los movimientos mandibulares deben realizarse adecuadamente mediante guía anterior o función grupal para no afectar la articulación temporomandibular.

**Figura 3:**
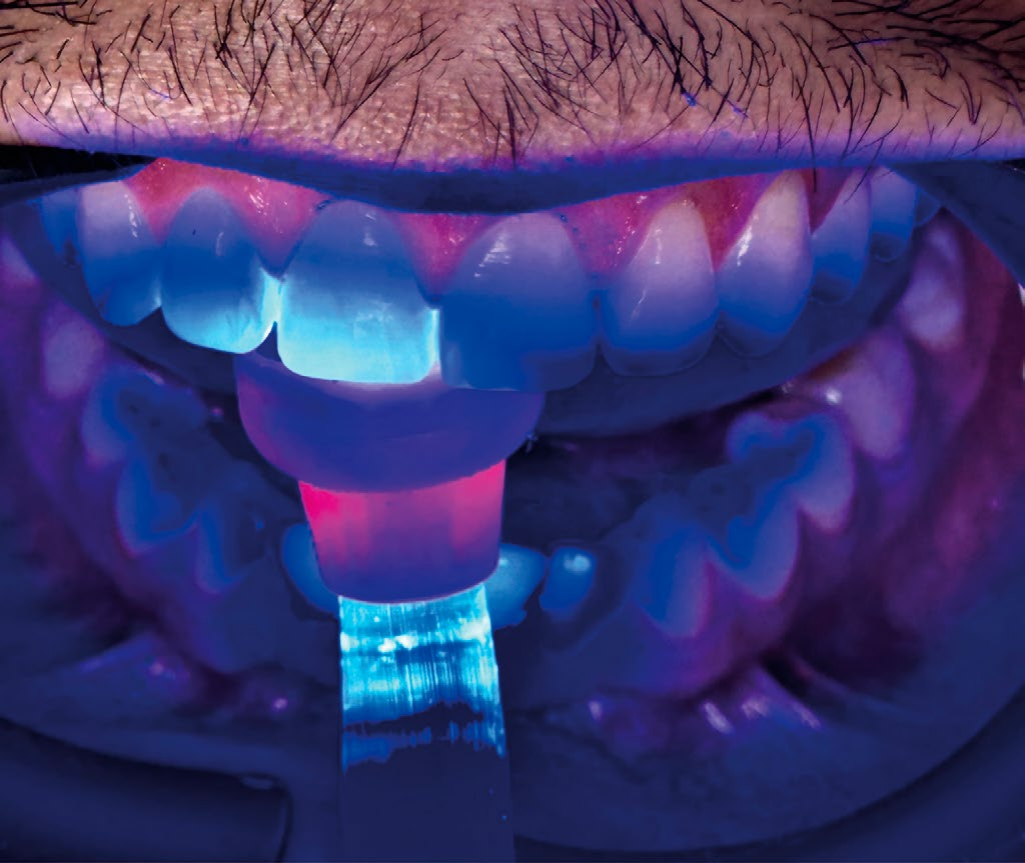
Luz de unidad de fotocurado sobre el esmalte seco

**Figura 4:**
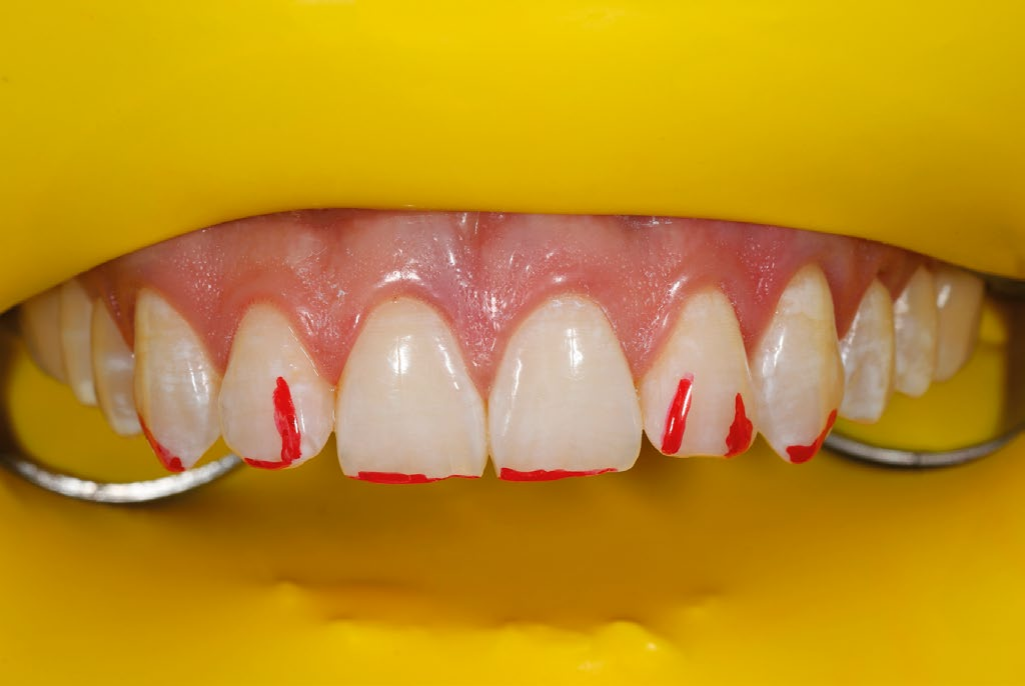
Áreas marcadas con lápiz rojo para reducción selectiva del esmalte.

Para evaluar la calidad y cantidad del esmalte mediante transiluminación se utilizó una unidad de fotopolimerización sobre esmalte seco, con el fin de establecer la proyección de la reducción selectiva del esmalte. La luz se llevó a 3 posiciones: posición 1: luz sobre el cíngulo de los dientes anterosuperiores; posición 2: luz sobre el tercio medio de la superficie palatina; y posición 3: luz sobre el borde incisal en el esmalte seco (Fig. 3). La mejor posición para evaluar el espesor del esmalte es la posición 1. Cuando se lleva la luz desde la zona cérvico-palatina hacia el borde incisal, se pueden identificar las zonas de reducción selectiva del esmalte para marcarlas con un lápiz rojo (Fig. 4). Una vez identificadas, se toma una fotografía para registrar las marcas y usar esa imagen durante el procedimiento.

**Figura 5:**
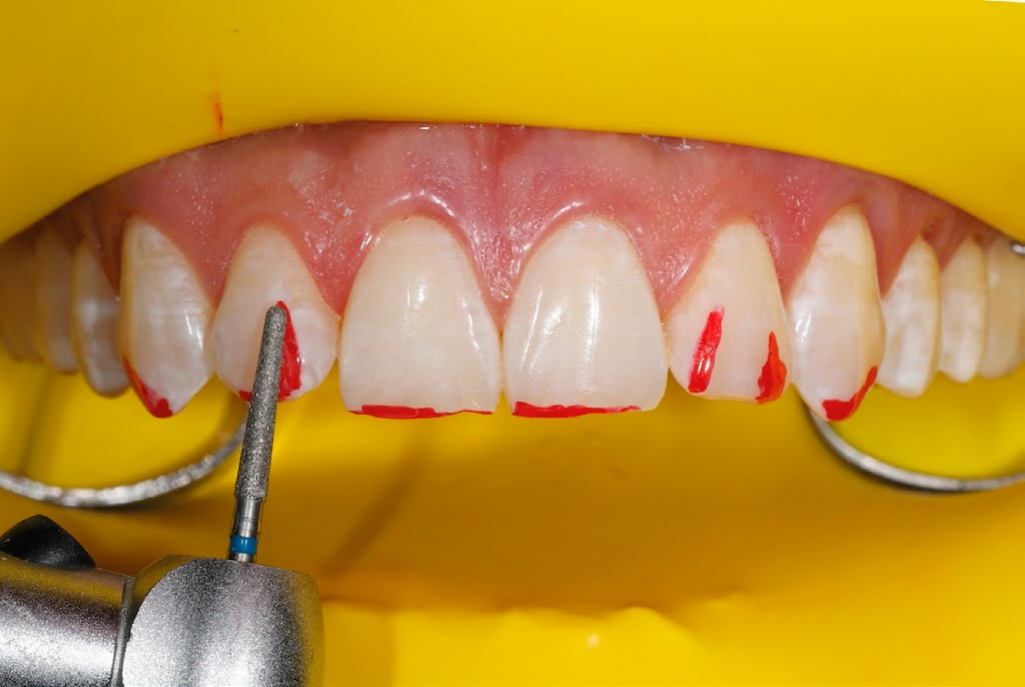
Reducción del esmalte con fresa de diamante de punta redondeada.

Para iniciar la reducción selectiva del esmalte, se debe empezar con una fresa abrasiva y, luego, se emplea una fresa diamantada de grano medio con punta redondeada en las zonas marcadas con irrigación abundante (Figs. 5 y 6). El proceso se inicia en los incisivos centrales, luego se pasa a los laterales, los caninos y, finalmente, los premolares. El paciente debe sentarse para pedirle que haga movimientos, hable y sonría, a fin de analizar el nuevo contorno y los diferentes ángulos, como el facial, labial y dental. Después, se continúa usando una fresa de diamante de grano extrafino (Fig. 7), con la cual se pulen las zonas tratadas, teniendo en cuenta que los ángulos interincisales deben ser redondeados o dejarlos rectos, según conveniencia estética. 

**Figura 6:**
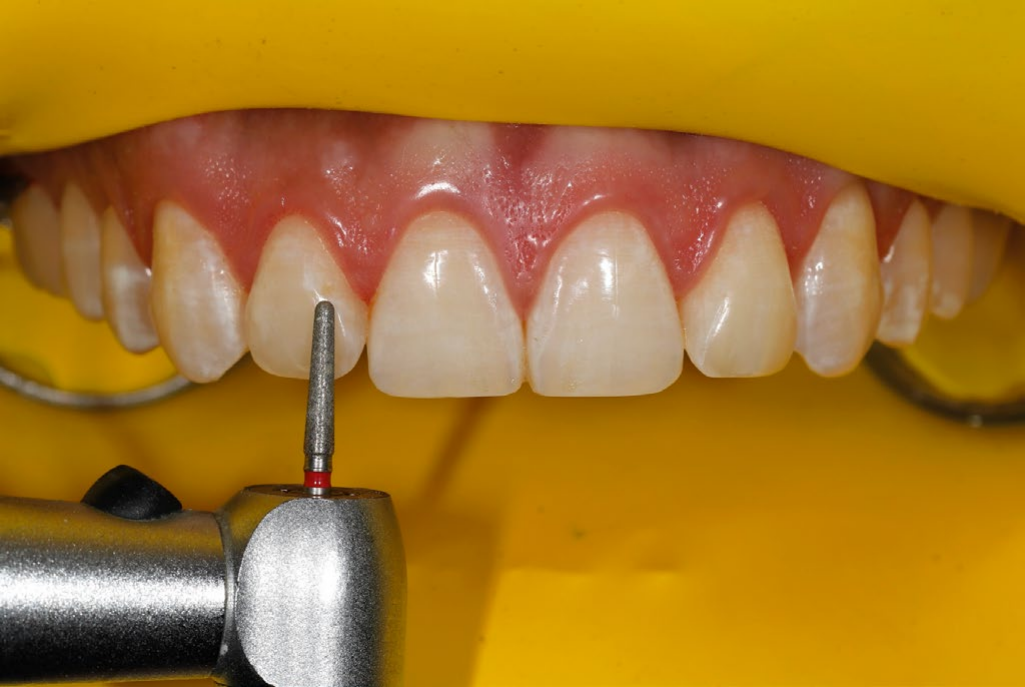
Reducción del esmalte con fresa de diamante de grano medio de punta redondeada.

**Figura 7:**
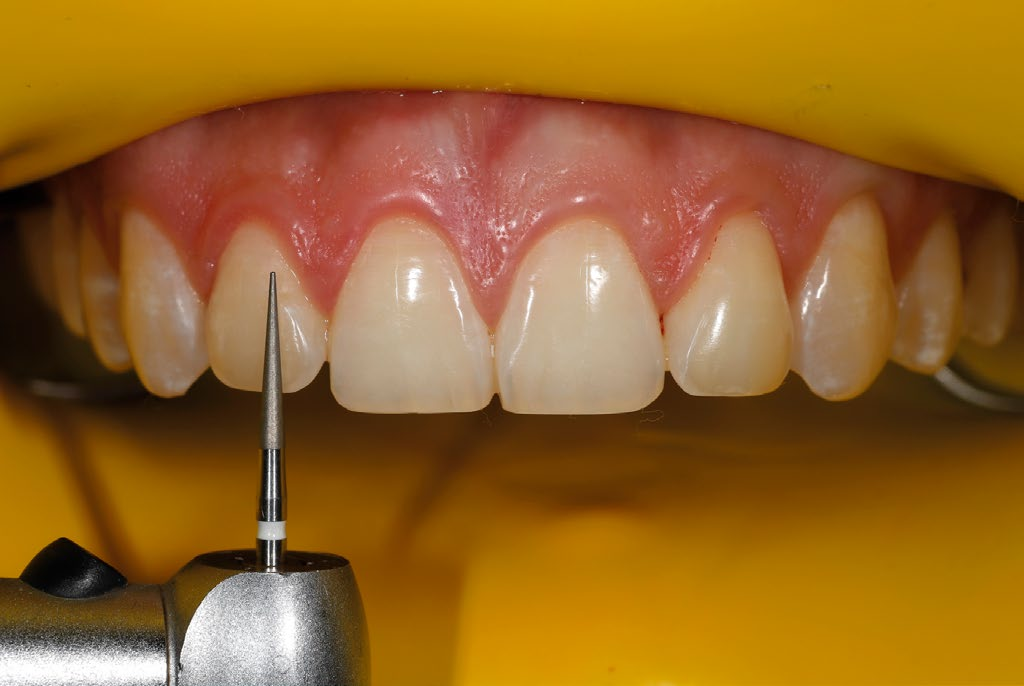
Reducción del esmalte con fresas de diamante de grano fino y extrafino.

**Figura 8:**
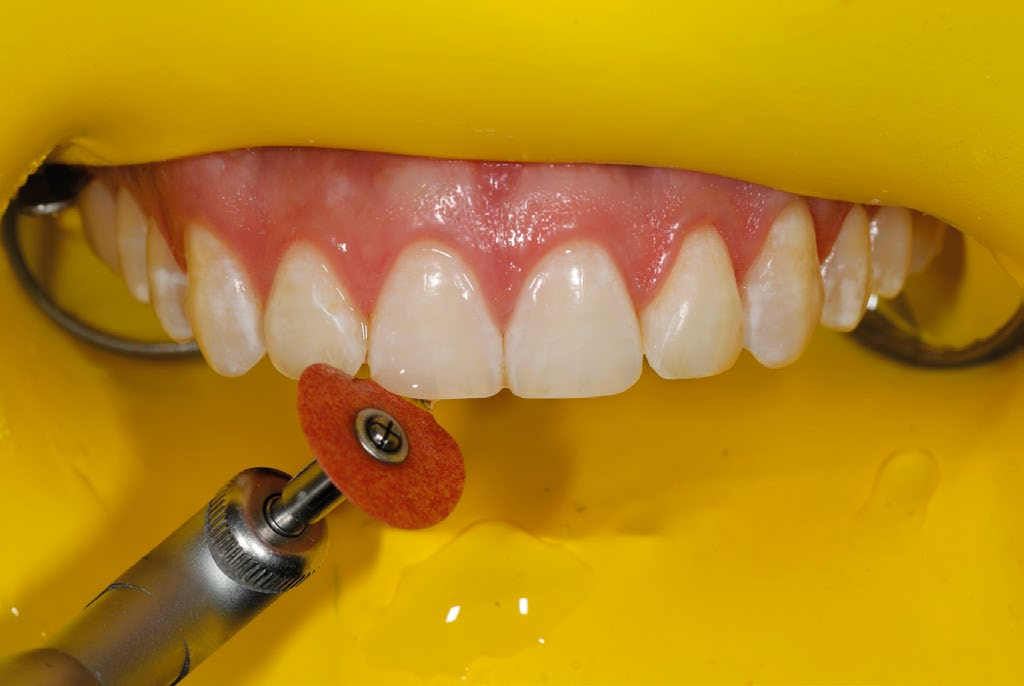
Pulido de superficie con discos Sof-Lex.

Una vez más, se sienta al paciente y, en esta posición, se usan los discos Sof-Lex, primero el más abrasivo, para ajustar la forma y el contorno anatómico a los dientes que lo requirieron. Todo esto se realiza en simultáneo con un análisis de los movimientos labiales y la gesticulación. Se continúa con el Sof-Lex de grano medio y fino, siempre con abundante irrigación con agua (Fig. 8). 

**Figura 9:**
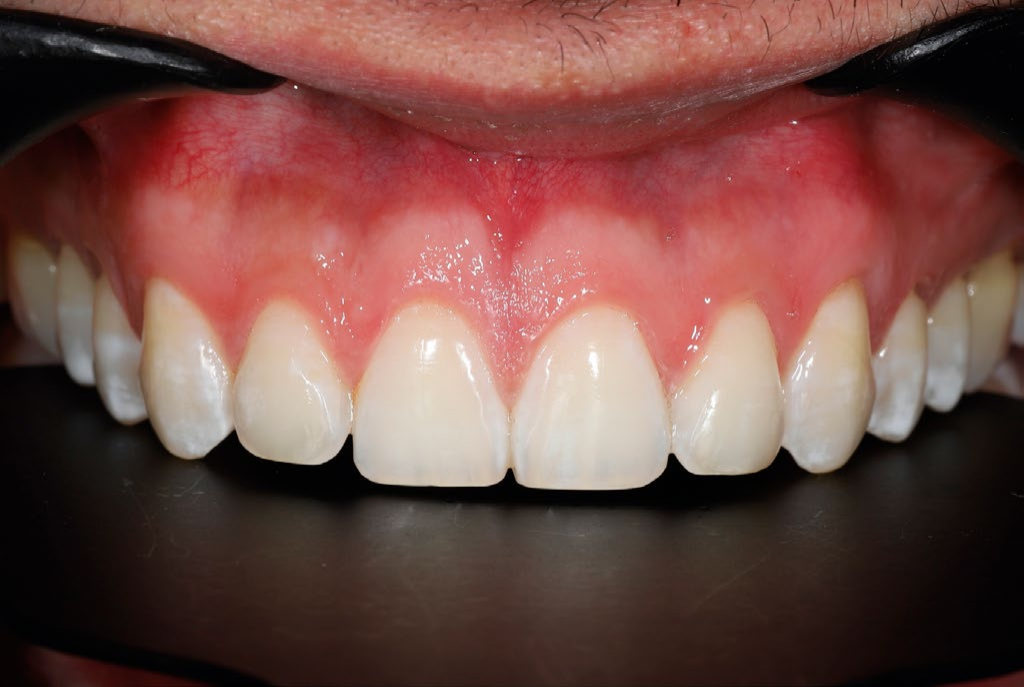
Resultado final

El proceso de remineralización del esmalte se lleva a cabo mediante métodos naturales y artificiales. El actor principal es la saliva, con sus proteínas que nos ayudan de forma natural a recuperar el esmalte y, artificialmente, el flúor neutro en las zonas tratadas, para finalmente recomendar el uso de pasta dental desensibilizante como complemento. Así se logra un alto resultado estético (Fig. 9).

## DISCUSIÓN

La reducción del esmalte es un procedimiento clínico que implica un remodelado anatómico y la protección de las superficies del esmalte de los dientes permanentes. Desde 1944, cuando Ballard utilizó por primera vez la reducción del esmalte interproximal en el segmento anterior, se han creado muchas técnicas diferentes para ganar espacio en el arco dental, por ejemplo, las de Hudson (1956), Paskow (1971), Peck y Peck (1972), el método de *air-rotor stripping* (ARS) de Sheridan (1985) y el método de Zachrisson (1986) ^9^.

El recontorneo de los dientes por medio de la reducción del esmalte puede ser, en ocasiones, un paso más dentro del plan de tratamiento estético, debido a las anormalidades en la forma de los dientes. 

En 2016, Mathias *et al*. ^10^ usaron la técnica de desgaste selectivo del esmalte para recontornear los caninos superiores y mejorar el aspecto de la sonrisa. A diferencia de este reporte de caso, ellos realizaron un complemento en algunas áreas de los caninos con resina compuesta, lo cual demuestra que es una alternativa a tratamientos más invasivos y tiene menor costo.

Con frecuencia, las restauraciones indirectas son una opción adecuada; sin embargo, el desarrollo de las resinas compuestas y los sistemas adhesivos permitieron un tratamiento alternativo mediante reconstrucciones directas usando resina ^11^. Este reporte de caso presenta el recontorneo de los dientes anteriores a través de la técnica de reducción selectiva del esmalte como un método para alcanzar excelentes resultados estéticos con una mínima intervención, sin el uso de materiales dentales restauradores, lo que representa algunas ventajas en comparación con las restauraciones tradicionales, como las carillas cerámicas o en resina, que podrían presentar complicaciones como pigmentación marginal, fractura, delaminación, caries secundarias o necesidad de tratamiento endodóntico ^12^^,^^13^. 

No se ha encontrado evidencia de antecedentes sobre técnicas similares que utilicen exclusivamente la reducción del esmalte en múltiples dientes de manera simultánea con el objetivo de mejorar la apariencia. Por otra parte, el desgaste selectivo del esmalte ha sido usado con mayor frecuencia en superficies interproximales para mejorar los objetivos de la ortodoncia, e incluso es usado en la mayoría de los casos con la técnica de alineadores, con lo que se convierte en una herramienta muy útil para los casos de apiñamiento dental ^14^^,^^15^.

El recontorneo cosmético se puede realizar de manera aditiva y de manera sustractiva, como en el caso presente. Esta segunda opción es recomendada por sus resultados inmediatos y permanentes. Adicionalmente, se presenta como una alternativa valiosa a tratamientos más costosos que demandan una mayor técnica operatoria. El recontorneo puede proporcionar un tratamiento rápido y eficiente en los pacientes para quienes está indicado ^16^. Por ejemplo, pacientes posadolescentes con oclusión borde a borde en la dentición permanente, con apiñamiento moderado y perfil ortognático, tanto con extracción como no extracción, el recontorneo por reducción parece producir resultados exitosos ^8^^,^^16^^,^^17^.

## CONCLUSIÓN

Esta técnica de recontorneo basado en reducción selectiva del esmalte ofrece ventajas como resultados inmediatos con beneficios estéticos y funcionales que pueden considerarse económicos y seguros comparados con los métodos convencionales.

## Fuente de financiamiento:

Ninguno
